# Editorial: Tumor microenvironment in cancer hallmarks and therapeutics

**DOI:** 10.3389/fmolb.2022.1019830

**Published:** 2022-09-12

**Authors:** Na Luo

**Affiliations:** Department of Anatomy and Histology, School of Medicine, Nankai University, Tianjin, China

**Keywords:** tumor microenvironment, non-coding RNA, tumor immune microenvioronment, cancer biomarkers, cancer signature

## Introduction

Tumor cells reside in an acidic, hypoxic, immunosuppressive, and nutrition-deficient environment called the tumor microenvironment (TME) which reprograms both metabolism and signaling pathways to support the uncontrolled proliferation of tumor cells. The TME features blood and lymphatic vessels, stromal cells, immune cells, and an extracellular matrix (e.g., cytokines, chemokines, collagen, proteoglycans, etc.). The dynamic and reciprocal communication between the TME and tumor cells contributes to survival, proliferation, invasion, metastasis, angiogenesis, drug resistance, and immune evasion of the tumor. This manuscript focuses on the TME contributions to cancer progression and TME targets with the potential for pharmaceutical intervention in cancer treatment.

## Non-coding RNA and cancer development

Many studies have focused on the connection between non-coding RNAs and cancer development. Non-coding RNAs are multifunctional molecules that interact with RNA, DNA, or proteins to either promote or prevent the expression of protein-coding genes (Anastasiadou et al., 2018). Non-coding RNAs are dysregulated in tumor cells as well as stromal cells which provides a novel perspective for the regulation of TME components (Di Agostino et al., 2022). Moreover, studies have shown that RNA N6-methyladenosine (m6A) modification plays a substantial role in TME diversity and complexity (Li et al., 2021; Si et al., 2021).

Zheng et al. reviewed the function, mechanism, and clinical significance of lymph enhancer-binding factor 1-antisense RNA1 (LEF1-AS1) which plays a dual-edged role in cancer development. In this regard, LEF1-AS1 functions either as a tumor suppressor in myeloid malignancy or a tumor promoter in other malignant tumors. Wang et al. reviewed the relationship of non-coding RNA expression and the influence of *H. pylori* on gastric cancer which provided a new perspective for gastric cancer treatment.

Furthermore, Tu et al. demonstrated that circ_0021205 promotes proliferation, migration, and invasion of cholangiocarcinomas (CCA) cells in addition to tumorigenesis in mice via sponging miR-204-5p which regulates RAB22A expression. This suggests that circ_0021205 may be a diagnostic biomarker or therapeutic target for CCA. Furthermore, Chen et al. showed that upregulation of miRNA-7062-5p promotes osteoclast genesis during bone metastasis via GPR65 inhibition. Consequently, we can postulate that the miRNA-7062-5p/GPR65 axis may serve as a therapeutic target for colorectal cancer (CRC) metastasis to the bone.

In addition, many studies have used comprehensive bioinformatics analysis to investigate the association of non-coding RNA and the TME, especially the immune environment of TME. Huang et al. analyzed the influence of the stromal, immune, and estimate scores on the prognosis of hepatocellular carcinoma (HCC) patients. They established a novel TME-related lncRNA risk model which may serve as an independent prognostic biomarker and predictor of immune checkpoint inhibitors (ICIs) for HCC patients. Xiao et al. showed that LINC02257 is the most significantly survival-associated enhancer RNA (eRNA) whose expression correlates with a multi-omics analysis of 33 cancer types, such as survival analysis and immunotherapy-related analysis. These findings suggest that LINC02257 is a multifaceted and important immunotherapy-related eRNA in different cancers. Ma et al. showed that the LINC00987/A2M axis is a functional and effective tumor suppressor of lung adenocarcinoma (LUAD). The LINC00987/A2M axis functions as a biomarker either for assessing the immune microenvironment or evaluating the prognostic and therapeutic potential of LUAD. In addition, Ren et al. proposed that a novel competing endogenous RNA (ceRNA) network modulates genomic integrity and demonstrated that a genome instability-related lncRNA serves as a novel prognosis biomarker for immunotherapy outcomes of colon adenocarcinoma (COAD). Moreover, Guo et al. analyzed the differential genomic instability-associated lncRNAs between left-sided and right-sided colon cancers (LCCs and RCCs) and identified six key DGIA lncRNAs. These six key DGIA lncRNAs not only predict the prognostic risk of patients but also serve as biomarkers for evaluating differences of genetic instability, immune infiltration, and therapeutic sensitivity.

m6A RNA modifications are reversible epigenetic RNA modifications that modulate splicing, degradation, and other biological processes of RNAs. m6A RNA modifications of non-coding RNA are associated with tumorigenesis, metastasis, and other tumor characteristics (Li et al., 2021; Si et al., 2021). Specifically, Jing et al. summarized the biological function, mechanism, and clinical significance of m6A RNA modifications in head and neck squamous cell carcinoma (HNSCC) from a systematic perspective. The authors thoroughly discussed the regulatory roles and potential molecular mechanisms of m6A in immune cells in the TME and the development of potential targets for treating HNSCC. Xu et al. comprehensively analyzed m6A RNA methylation regulators in HCC and revealed that m6A regulators were significantly associated with the tumor immune microenvironment in HCC. This finding may provide a new prognostic biomarker and therapeutic target for improving the efficacy of immunotherapy in HCC patients.

## Tumor immune microenvironment and cancer development

Immune checkpoint inhibitor (ICI) therapy has revolutionized the field of cancer treatment. Despite the remarkable and substantial effects of ICIs on cancer patients, only a small percentage of patients achieve clinical benefits (Jacob et al., 2021). In this regard, the ability to increase the response rates and overcome ICI resistance is the key to improve clinical efficacy.

A prevalent strategy to increase the response rate of ICIs involves combination therapy with potential pharmaceutical targets and ICIs. For example, Fu et al. employed a novel combination therapy utilizing foretinib (a multiple receptor tyrosine kinase inhibitor) and anti-PD-1 antibody for CRC treatment. The combination therapy significantly inhibited tumor growth in mice by increasing PD-L1 expression via JAK2/STAT1 pathway. Simultaneously, combination therapy remodeled the tumor microenvironment by: 1) increasing the infiltration and function of T cells, 2) decreasing the percentage of tumor-associated macrophages (TAMs), and 3) inhibiting M2 polarization. Moreover, combination therapy inhibited metastasis to the lung and remodeled the tumor microenvironment in the lung. Wang et al. discovered that combination therapy with metformin and PD-1 inhibitor enhanced anti-tumor efficacy in STK11 mutant lung cancer. The metformin and PD-1 inhibitor combination therapy involved the inhibition of RNF5-mediated K48-linked ubiquitination of STING and showed AXIN-1 dependence.

The tumor immune microenvironment (TIME) consists of cancer cells, immune cells, cytokines, chemokines, etc. And potentially influences cancer development. Remodeling the TIME is a popular strategy to impede cancer development. Li et al. reviewed the role of immune cells in the crosstalk between the TME and cancer cells and the effects of ICIs therapy on these cell populations. The authors also discuss the potential of the functional interaction between TME and cancer cells as a predictive biomarker for ICIs, and outline the potential personalized strategies to improve the effectiveness of ICIs. Peng et al. found that lung adenocarcinoma cells promote self-invasion and self-migration by activating neutrophils to upregulate the Notch3 expression of cancer cells. In this regard, the density of infiltrating tumor-associated neutrophils (TANs) may serve as a novel prognosis biomarker and a potential therapeutic target for lung adenocarcinoma. In addition, Li et al. reviewed the favorable TME conditions that promote recruitment, expansion, activation, and immunosuppression of myeloid-derived suppressor cells (MDSCs) and also discussed the precision targeting MDSCs for therapeutic intervention.

Xiong et al. reviewed the role of chemokine CXCL8 in directly promoting or indirectly facilitating tumor progression and also role of CXCL8 in ICI therapy. The preclinical studies suggest combinational therapy with CXCL8 blockade and ICIs therapy can enhance the anti-tumor efficacy, and the findings promote conduction of several clinical trials. Moreover, Liu et al. showed that SB225002 (a CXCR2 inhibitor) promotes antitumor and radio-sensitization effects in nasopharyngeal carcinoma (NPC). The authors proposed that the CXCL8/CCR2 axis functions as a prognosis biomarker for NPC patients and serves as a promising therapeutic target for NPC treatment. Chen et al. demonstrated that interferon-inducible protein 16 (IFI16) adversely correlates with the overall survival of pancreatic adenocarcinoma (PAAD) patients and promotes PAAD progression via IL-1β-induced TAMs infiltration. These findings suggest that IFI16 may function as a potential therapeutic target for PAAD.

Finally, Liu et al. compiled a list of immune genes and immune infiltrating cells that play a role in the prognosis of HNSCC. This list provides a valuable roadmap to assess HNSCC evolution and treatment selection. Gong et al. investigated the immune cell infiltration (ICI) landscape of thyroid cancer (THCA) and showed that the ICI score is an effective prognostic indicator and predictor of immunotherapy response in THCA.

## Metabolism and TME

Metabolic factors are not only crucial for tumor growth, but also for remodeling the TME to produce a favorable environment for tumor invasion and metastasis. This metabolic adaptation requires the re-adjustment of metabolic pathways and signaling pathways that permits the import/consumption of organic compounds and subsequent energy and biomass production (Serpa, 2020).

Qiu et al. clarified the metabolic properties of cancer cells in glioma and the interactions with TME immunity. The authors also discuss the therapy strategies targeting metabolic remodeling to improve glioma immunity.

Li et al. found that the expression of phosphoglycerate kinase 1(PGK1) [a glycolytic enzyme that catalyzes the conversion of 1,3-diphosphoglycerate to 3-phosphoglycerate] was upregulated in various types of breast cancer. In this regard, PGK1 expression significantly associates with survival signature and correlates with TP53 and CDH1 mutations. These results indicate that PGK1 may serve as a potential biomarker for breast cancer.

Moreover, Hao et al. demonstrated that oxysterol binding protein like 3 (OSBPL3) which belongs to a group of intracellular lipid receptors plays an important role in tumorigenesis via comprehensive analyzation of publicly available databases of various cancers. These results provide insights into the biological functions of OSBPL3. Furthermore, Liu et al. performed comprehensive analysis and reported that hexokinase 2 (HK2) [an enzyme that phosphorylates glucose to produce glucose-6-phosphate in the first step of glucose metabolism] is associated with tumor infiltration and m6A modification in esophageal carcinoma (ESCA). This suggests that HK2 may serve as a potential biological target for diagnosis and treatment in ESCA.

## Nutrition and TME

Nutritional stimuli modulate the homeostasis of tumor cells and TME which then leads to a consequent alteration of tumorigenesis and tumor progression (Thakkar et al., 2022).

Li et al. performed a retrospective study to investigate the prognostic nutritional index (PNI) calculated as serum albumin (ALB) (g/L) + 5x total lymphocyte count (109/L). They found that PNI before neoadjuvant chemotherapy (NACT) served as a useful prognostic indicator and as a promising biomarker for treatment decisions in breast cancer patients.

Yang et al. discovered that a fucoidan-supplemented diet improves the anti-tumor activities of PD-1 antibodies *in vivo*. The mechanism involves cooperation with the JAK/STAT pathway and interaction with the T-cell receptor (TCR)/CD3 complex in order to stimulate T cell activation and augment TCR-mediated signaling. Moreover, Shi et al. showed that TR35 (an active camel whey fraction) plays a role in NSCLC progression via MAPK and JAK/STAT pathway. This finding indicates that TR35 may serve as a potential therapeutic agent in lung cancer.

## Cancer-associated fibroblasts or tumor-associated fibroblasts

CAFs or TAFs are hallmark features of the TME and promote cancer/tumor progression. In this regard, Deng et al. summarized the origins, biomarkers, prognostic significance, functional roles, and underlying mechanisms of CAFs in the colorectal cancer (CRC) and discussed harnessing CAFs as promising therapeutic targets for CRC treatment. Moreover, Ji et al. found that IL-8 secretion from CAFs stimulated malignant growth and induced the stemness of ovarian cancer via the Notch3 signaling pathway which may provide a novel strategy for ovarian cancer treatment. In addition, Zheng et al. constructed a 4-gene prognostic CAF model which predicts prognosis and estimates the immunotherapy response in gastric cancer (GC) patients.

## Pathways related to cancer development

It is well-established that cancer is caused by the dysregulation of pathways involved in cell survival, proliferation, migration, differentiation, and apoptosis (Yip and Papa, 2021). Zhang et al. describe the role of PI3K in breast cancer progression and resistance in currently used treatments. This minireview also addressed the clinical application of PI3K inhibitors and the potential synergistic benefit of combined PI3K/immunotherapy in breast cancer treatment. Zhu et al. extensively reviewed the expression and function of complement pathway components in multiple tumor types which may serve as prognostic factors or therapeutic targets in malignant glioma treatment.

Tang et al. reviewed the role of the androgen receptor (AR) in cross-talk between stromal cells and prostate cancer cells and discussed the development of novel therapeutic strategies targeting the AR pathway. Liu et al. reviewed the role of ferroptosis within the TME and its influence on cancer development and progression. The authors also discuss the novel therapeutic strategies targeting ferroptosis-related pathways and metabolism for cancer treatment. Furthermore, Guo et al. verified that PPA1 activates PI3K/AKT signaling and promotes breast cancer progression via the downstream GSK3β/Slug signaling. This verification suggests that PPA1 may serve as a potential therapeutic target to inhibit breast cancer progression.

## Large-scale data mining and bioinformatics analysis with cancer signature

A gradually emerging phenomenon with the development of large-scale data mining and bioinformatics analysis is the establishment of immune, survival, or prognosis signatures which boost the development of precision medicine or individualized therapies.

Qian et al. constructed an immune-related 18 gene signature that predicts which PDAC subtype may benefit from gemcitabine-based adjuvant chemotherapy or may respond to PD1/PD-L1 blockade therapy. Zhou et al. constructed an immune-related gene signature of the TME landscape which may serve as a prognostic biomarker or as a predictor of an immunotherapy effect in skin cutaneous melanoma (SKCM). Wu et al. developed a TAM migration/transformation-related three gene signature which may serve as a prognostic biomarker and as a guide to individualized treatments in LUAD. Mao et al. constructed an adhesion-related 18 gene signature leading to the formation of the focal adhesion index (FAI) which may serve as a prognostic biomarker of the immune microenvironment in GC. Liang et al. constructed two immune-related genes (IRG)-relate prognostic signatures based on gene-immune interaction for predicting risk stratification and immunotherapeutic responses, which may be helpful to screen the people who will benefit from immunotherapy and guide the clinical decision-making of patients with bladder urothelial carcinoma (BLCA).

In addition to the above-mentioned immune-signature models, Feng et al. developed a 7-gene prognostic signature to improve survival prediction, individualized therapy, and appropriate management in PDAC patients. Li et al. established two survival subtypes of oral squamous cell carcinoma (OSCC) using a deep learning approach that provides novel precision-medicine treatment options and improves survival times in OSCC patients. Yang et al. established a novel skin cutaneous melanoma (SKCM) classification based on metabolic gene expression profiles to understand the metabolic diversity of SKCM and to provide guidance on precision targeted therapy. Liu et al. identified an oxidative phosphorylation (OXPHOS)-related signature which classifies uterine corpus endometrial carcinoma (UCEC) patients into different risk subsets and predicts prognosis.

## Cancer biomarkers

Bioinformatics analysis of “omics” data provides a route to identify reliable cancer biomarkers for cancer diagnosis and prognosis and to discover molecular targets for therapeutic intervention (Feltes et al., 2020; Li et al., 2020). Jiang et al. found that the expression levels of cell division cycle associated (CDCA) 1/3/5/8 correlate with poor prognosis and may serve as a diagnostic gene in hepatocarcinogenesis and as a prognostic biomarker in hepatocellular carcinoma (LIHC) patients. Li et al. revealed that p-JNK effectively predicts the survival in breast cancer patients receiving NACT. Moreover, they found that anisomycin (a JNK agonist) increases p-JNK expression and may benefit breast cancer patients receiving NACT. In addition, Zheng et al. showed that CALD1 upregulation occurs in the CMS4 CRC subtype which is associated with angiogenesis and TGF-β signaling gene sets in stage III/IV mismatch repair-proficient (pMMR) CRC samples using multiple bioinformatic analyses and cell-level assays. CALD1 also associates with immune and stromal components in the TME (e.g., antigen processing and presentation, chemokine/cytokine signaling). This suggests that CALD1 may serve as a prognostic biomarker and a therapeutic target for stage III/IV pMMR CRCs. Liu et al. identified a CDK2-related immune forecast model, a Nomogram model, a forest map, and a ceRNA network that may predict the prognosis and guide targeted therapy in LUAD patients utilizing a pan-cancer analysis. Yu et al. found that cAMP response element modulator (CREM) may serve as a prognostic biomarker in gastric adenocarcinoma (GAC) using integrated bioinformatics methods. Wang et al. showed that secreted modular calcium-binding protein 1 (SMOC1) influences the progression on glioma and may serve as a prognosis biomarker for glioma using multiple databases.

Besides bioinformatics analysis, Tan et al. showed that higher MALT1 expression levels occur in cancer samples versus normal tissues. MALT1 promotes proliferation, colony formation, and cancer establishment. Moreover, MALT1 expression is closely related to the occurrence and development of multiple cancers which suggest that MALT1 may serve as therapeutic target for a variety of cancers. Furthermore, Xia et al. reviewed the roles of Ikaros (a zinc finger transcription factor) in tumorigenesis and drug resistance, and discussed targeting Ikaros as a treatment either in single or combination use.

## Clinical consideration and cancer development

Zhao et al. explored the dynamic changes in the TME after hypo-fractionated radiotherapy (HFRT) in NSCLC and established the timing and fractionation dose for achieving the optimal immune response. Han et al. revealed that the combination of radiomic (such as MRI scanning) and non-radiomic practices are helpful to differentiate among gliobastoma multiforme (GBM), metastasis (MET)-lung, and MET-other. Interestingly, Lou et al. found that tumor purity might be a patient-specific intrinsic characteristic of GC which associates with survival time and recurrence. Tumor purity is also associated with an invasive, metastatic phenotype along with immune and stromal cell functions. These findings highlight that tumor purity confers important clinical, biological, micro-environmental, and treatment implications for patients with GC.
